# MSC-derived mitochondria promote axonal regeneration via *Atf3* gene up-regulation by ROS induced DNA double strand breaks at transcription initiation region

**DOI:** 10.1186/s12964-024-01617-7

**Published:** 2024-04-25

**Authors:** Yingchi Zhang, Tao Xu, Jie Xie, Hua Wu, Weihua Hu, Xuefeng Yuan

**Affiliations:** 1 Department of Traumatology, Tongji Hospital, Tongji Medical College, Huazhong University of Science and Technology, Jiefang Avenue 1095, Wuhan, Hubei, 430030 People’s Republic of China; 2 Department of Orthopedics, Tongji Hospital, Tongji Medical College, Huazhong University of Science and Technology, Jiefang Avenue 1095, Wuhan, Hubei, 430030 People’s Republic of China

**Keywords:** Peripheral nerve injury, Axonal regeneration, Bone marrow mesenchymal stromal cell, Activating transcription factor 3, DNA double strand break

## Abstract

**Background:**

The repair of peripheral nerve injury poses a clinical challenge, necessitating further investigation into novel therapeutic approaches. In recent years, bone marrow mesenchymal stromal cell (MSC)-derived mitochondrial transfer has emerged as a promising therapy for cellular injury, with reported applications in central nerve injury. However, its potential therapeutic effect on peripheral nerve injury remains unclear.

**Methods:**

We established a mouse sciatic nerve crush injury model. Mitochondria extracted from MSCs were intraneurally injected into the injured sciatic nerves. Axonal regeneration was observed through whole-mount nerve imaging. The dorsal root ganglions (DRGs) corresponding to the injured nerve were harvested to test the gene expression, reactive oxygen species (ROS) levels, as well as the degree and location of DNA double strand breaks (DSBs).

**Results:**

The in vivo experiments showed that the mitochondrial injection therapy effectively promoted axon regeneration in injured sciatic nerves. Four days after injection of fluorescently labeled mitochondria into the injured nerves, fluorescently labeled mitochondria were detected in the corresponding DRGs. RNA-seq and qPCR results showed that the mitochondrial injection therapy enhanced the expression of *Atf3* and other regeneration-associated genes in DRG neurons. Knocking down of *Atf3* in DRGs by siRNA could diminish the therapeutic effect of mitochondrial injection. Subsequent experiments showed that mitochondrial injection therapy could increase the levels of ROS and DSBs in injury-associated DRG neurons, with this increase being correlated with *Atf3* expression. ChIP and Co-IP experiments revealed an elevation of DSB levels within the transcription initiation region of the *Atf3* gene following mitochondrial injection therapy, while also demonstrating a spatial proximity between mitochondria-induced DSBs and CTCF binding sites.

**Conclusion:**

These findings suggest that MSC-derived mitochondria injected into the injured nerves can be retrogradely transferred to DRG neuron somas via axoplasmic transport, and increase the DSBs at the transcription initiation regions of the *Atf3* gene through ROS accumulation, which rapidly release the CTCF-mediated topological constraints on chromatin interactions. This process may enhance spatial interactions between the *Atf3* promoter and enhancer, ultimately promoting *Atf3* expression. The up-regulation of *Atf3* induced by mitochondria further promotes the expression of downstream regeneration-associated genes and facilitates axon regeneration.

**Supplementary Information:**

The online version contains supplementary material available at 10.1186/s12964-024-01617-7.

MSC-derived mitochondria promote axonal regeneration via *Atf3* gene up-regulation by ROS induced DNA double strand breaks at transcription initiation region.

## Introduction

Axonal injury to sensory and motor neurons in the peripheral nervous system (PNS) can regenerate completely, whereas in the central nervous system (CNS) cannot [1–6]. A major reason for this difference is that the gene expression within PNS neurons changes significantly after axonal injury, enabling them to transition from a state of quiescence to a state of active growth or regeneration. *Atf3, Ap1, Jun, Creb, Stat3, Nf-kb,, Sox11* and other transcription factor coding genes were involved in the early injury response within a few hours after nerve injury [7–11]. These early response genes then co-regulate the expression of later regenerative effect genes in a specific manner [9, 12]. Although this injury response can lead to successful axon regeneration in a mouse model of peripheral nerve injury [13, 14], the length of the human nerve is significantly longer than that of the mouse nerve. The extended regeneration distance may impede the seamless growth of the regenerated nerve towards the peripheral target organ [15–17]. Therefore, clinical patients with peripheral nerve injuries may struggle to achieve complete functional recovery following surgical treatment. New therapies aimed at enhancing or accelerating the regeneration of peripheral nerve injuries will play a crucial role in restoring limb function in patients with nerve injuries, which has significant clinical value and warrants further exploration.

Communication and transportation of materials between nerve and supporting cells have been increasingly recognized as crucial factors in the repair of nerve injuries. Schwann cells (SCs), the glial cells of the PNS, are the primary supportive cells in the PNS. Following nerve damage, SCs undergo dedifferentiation and initiate a reparative program to facilitate nerve regeneration [18]. SCs engage in various mechanisms to interact with the peripheral nervous system, including the release of neurotrophic factors [19] and transfer of extracellular vesicles [20], metabolites [21, 22], ions [23], and organelles [24, 25].

Although SCs play a pivotal role in the regeneration of peripheral nerves, their limited accessibility from patients hampers their potential for clinical application in cell therapy. Mesenchymal stromal cells (MSCs) are adult multipotent stromal cells that can be isolated from various human tissues such as adipose, bone marrow, umbilical cord blood and dental pulp [26]. MSCs have been identified as having multi-directional differentiation potential, high self-renewal ability, and low immunogenicity, making them one of the most common potential off-the-shelf stromal cells in cell therapy [27]. The efficacy of adult multipotent MSCs in promoting neuronal growth and survival has also been demonstrated by several studies, highlighting their potential for facilitating functional recovery following peripheral nerve injury [28–30]. However, there are several drawbacks to MSC-based therapy, including its high cost, cellular phenotypic instability, and the risk of microinfarction caused by transported MSCs becoming lodged in the pulmonary microvasculature [31, 32]. As a result, a new cell-free therapy with similar efficacy to that of MSCs must be developed for peripheral nerve injury.

Mitochondrial transfer has numerous therapeutic effects, such as supplying ATP to recipient cells, regulating reactive oxygen species (ROS), and buffering calcium ion concentration. In recent years, it has gradually emerged as a new therapy for cell injury [33–37]. In the central nervous system (CNS), astrocytes transfer mitochondria to injured neurons, promoting their survival and plasticity [38]. In spinal cord injury, MSCs can transfer mitochondria to motor neurons through tight junctions. The artificial transfer of purified mitochondria to the injured site can significantly reduce the apoptosis of motor neurons and promote spinal cord repair [39]. However, the use of mitochondrial transfer to treat peripheral nerve injury has not been extensively studied.

In our study, we constructed a mouse sciatic nerve crush injury model, and we injected MSC-derived mitochondria into the injured nerve. We found that the injected mitochondria transferred into the DRG neuron somas and promoted *Atf3* expression by accumulating ROS and triggering DNA double-strand breaks (DSBs) at the transcription initiation region of the *Atf3* gene. This process facilitated *Atf3* expression and accelerated axon regeneration. Furthermore, by studying the spatial relationship between DSB sites and CTCF proteins, we revealed that the mitochondria-induced DSBs were located in the vicinity of the CTCF binding site, which may lead to the release of the CTCF-induced topological constraints on the spatial interactions of the *Atf3* promoter and enhancer. This study will provide new insights into the application of mitochondrial transfer therapy in peripheral nerve injury.

## Materials and methods

### MSC culture

The 6th passage of bone marrow mesenchymal stromal cells (MSCs) derived from C57BL/6 mice was obtained from Cyagen Biosciences (Guangzhou, China). Following culture and upon reaching the 10th passage, the purchased MSCs were characterized by evaluating their surface markers indicative of MSC identity, as well as their multipotent potential to differentiate into adipogenic, osteogenic, and chondrogenic lineages (Figure S1A and B). The MSCs were cultured in Gibco Dulbecco’s modified Eagle medium/Ham’s F-12 (DMEM/F12) supplemented with 10% fetal bovine serum and 100 U/mL penicillin-streptomycin (Gibco, USA), under the conditions of 5% CO_2_, a temperature of 37℃, and a humidity level of 100%. Upon reaching approximately 90% confluence, trypsin at a concentration of 0.25% (Gibco, USA) was utilized to detach the cells for subsequent passaging at a ratio of 1:3. Passages ranging from 8th to 10th were employed for the following experimental procedures.

### Isolation of mitochondria from MSCs

Mitochondria were isolated from cultured MSCs using a Mitochondria Isolation Kit (#89,874, Mitochondria Isolation Kit for mammalian cells, Thermo Fisher, USA). The kit comprised three reagents (A, B, and C) specifically designed for isolating mitochondria. To isolate the mitochondria, the harvested cell suspension was centrifuged in a 2.0 mL microcentrifuge tube at 850 × g for 2 min to remove the supernatant. Subsequently, Reagent A was added to the MSCs in an ice-cooled tube. After vortexing at medium speed for 5 s and incubating on ice for 2 min, Reagent B was introduced into the tube, which was then kept on ice for 5 min. Vortexing at maximum speed every minute for a total of five times ensured proper mixing. Finally, Reagent C was added, followed by gently inverting the tube to mix thoroughly before centrifugation at 700 × g for 10 min at 4℃. The resulting supernatant containing mitochondria was carefully transferred to a new tube and subjected to further centrifugation at 12,000 × g for 15 min to harvest purified mitochondria. The structure and morphology of mitochondria were observed using a transmission electron microscopy (Figure S1C).

### Sciatic nerve injury (SNI) models

The study utilized male C57BL/6 mice at the age of 8 weeks, which were obtained from the Experimental Animal Center of Huazhong University of Science and Technology in Wuhan, China. Aseptic techniques were employed during the sciatic nerve crush surgeries while the mice were under 2.5% isoflurane anesthesia and analgesia. The right thigh of each mouse was surgically exposed at the mid-thigh level to access the sciatic nerve, which was subsequently crushed with moderate force using a forceps for 10 s, resulting in a crush size of 1 mm, equivalent to the width of the forceps tips (Figure S1D). For the SNI with mitochondrial injection therapy group, 2 µL of mitochondia (isolated from 10^6^ MSCs) were injected into the sciatic nerve at the crush site using a microsyringe (Figure S1E). Finally, a two-layer closure technique was used to close the surgical wound. Considering concerns about the potential influence on experimental results due to therapeutic factors associated with the administration of anti-pain drugs, we did not apply postoperative analgesics after the SNI modeling.

### Whole-mount staining of sciatic nerves

The sciatic nerves were fixed in 4% paraformaldehyde (PFA) overnight at 4℃. Fixed sciatic nerves were first dehydrated in an ascending serial concentration of tetrahydrofuran (50%, 70%, 80% and 100%, v/v % in distilled water, 20 min each, Sigma-Aldrich) and then cleared in a solution of benzyl alcohol and benzyl benzoate (BABB, 1:2, Sigma-Aldrich). Incubations were conducted on an orbital shaker at room temperature. The nerves were stored in BABB solution in the dark at room temperature. Following fixation and clearing, the nerves were washed in PTX (1% Triton X-100 (Sigma, T9284) in phosphate-buffered saline (PBS)) three times for 10 min each. Then, the nerves were subsequently incubated with a blocking solution (10% fetal bovine serum (FBS) in PTX) overnight at 4℃. The following day, nerves were incubated with primary antibodies against Tuj1 (1:200, #ab52623, Abcam) in PTX containing 10% FBS and incubated for 48 h at 4℃ with gentle vibration. After the incubation, the nerves were washed with PTX three times for 15 min each wash, followed by washing in PTX for 6 h at room temperature, with a change of PTX every 1 h. Secondary Alexa Fluor-conjugated secondary antibodies (1:500, #ab150077, Abcam) were diluted in PTX containing 10% FBS, and incubated with the nerves for 48 h at 4℃ with gentle rocking. Next, the nerves were washed in PTX three times for 15 min each, followed by washing in PTX for 6 h at room temperature, changing the PTX each hour, and then washed overnight without changing PTX at 4℃. When staining was complete, the nerves were mounted between a slide and a cover slip and compressed under 5 Kg of books overnight for imaging.

### Analysis of in vivo axon regeneration

To quantify the regeneration of axons in vivo, we initially captured fluorescent tiled images of a whole-mount segment of the sciatic nerve using a CCD camera connected to an inverted fluorescence microscope. The imaging process was controlled by Zeiss AxioVision software with the assistance of the MosaiX module. Subsequently, these tiles were stitched together to create a comprehensive image of the entire nerve segment. To determine their length, each identifiable axon stained with Tuj1 within this nerve segment was manually traced from the site where it was crushed to its distal axonal tip. We then compared the average length of the five longest traced axons in each individual nerve sample.

### CatWalk gait analysis

The CatWalk XT system (Noldus Inc., Netherlands) was used to assess gait recovery and motor function after SNI. This test involves monitoring each animal when it crosses a walkway with a glass floor illuminated along the long edge. Data acquisition was carried out using a high-speed camera, and paw prints were automatically classified by the software. The performance of each mouse was recorded three times, to obtain approximately 15 step cycles per mouse for analysis. Printed area and swing of each animal were obtained 4, 8, and 12 days after surgery.

### Atf3 knockdown in DRG neurons

*Atf3* knockdown was achieved in adult mouse dorsal root ganglions (DRGs) through the injection of siRNA targeting *Atf3* and in vivo electroporation. After anesthetizing the mice, a small surgical procedure was performed on the right side to expose the DRGs at the L3/4/5 level. Each DRG was injected with a microsyringe containing 0.1 nmol/1 µL of siRNA specifically targeting *Atf3*. Immediately following the injection, in vivo electroporation was conducted using a platinum tweezer electrode (BTX) connected to an ECM 830 Electro Square Porator (BTX), delivering five electric pulses (35 V, 15-ms duration, 950-ms interval). The incision site was then closed, and the mice were given a recovery period of 2–3 days before establishing the SNI model. The siRNAs used for *Atf3* knockdown and non-targeting control were sourced from Santa Cruz Biotechnology, Inc., Santa Cruz, CA (#SC-29,758 and #SC-37,007).

### RNA extraction and qRT-PCR analysis

DRGs were subjected to TRIzol reagent (Invitrogen, Carlsbad, CA, USA) for RNA extraction. The purity and concentration of the extracted RNA samples were determined by spectrophotometric analysis. Reverse transcription was performed using an EasyScript First-Strand cDNA Synthesis Super Mix kit (TransGen Biotech, Beijing, China) with 3 micrograms of RNA. Quantitative real-time PCR (qRT-PCR) was used to evaluate the relative mRNA expression levels of mouse *Atf3, Sox11, Lin28a, Gap43, Smad1, Lin28b, Jun* and *Gapdh* (Invitrogen), utilizing TransStart Eco Green qPCR Super Mix (TransGen Biotech). The Bio-Rad myiQ2 Sequence Detection System (Bio-Rad, Hercules, CA, USA) was employed in this study. Primers purchased from Invitrogen were utilized as per Supplementary Table S1 instructions while cycle conditions followed the specifications of the primers. Relative gene expression levels were normalized against *Gapdh* and *β-actin* and analyzed using the 2^−ΔΔct^ method.

### Western blot analysis

Proteins were isolated from DRGs using a RIPA buffer supplemented with protease inhibitor and phosphatase inhibitor (Boster Biol Tech). The lysates were centrifuged at 16,000 × g for 15 min at 4 °C, followed by storing of the collected supernatants at -20 °C until further analysis. To extract cytoplasmic and nuclear proteins, a Nuclear and Cytoplasmic Protein Extraction Kit (Beyotime, Shanghai, China) was employed following the manufacturer’s instructions. The protein concentration was determined using a BCA protein assay kit (Applygen, Beijing, China). Equal amounts of proteins from each sample were subjected to electrophoresis on a 10% SDS-PAGE gel (Bio-Rad), followed by transfer onto polyvinylidene fluoride (PVDF) membranes (Merck Millipore, Billerica, MA, USA). For membrane blocking purposes at room temperature for one hour, a solution containing 5% BSA in 1× TBST (0.1% Tween-20) was utilized. Following blocking, the membranes were exposed to primary antibodies (Cell Signaling Technologies Inc., Milan, Italy) targeting ATF3, γH2AX, 53BP1, CTCF (all at a dilution of 1:1000), and β-actin (at a dilution of 1:400) overnight at 4 °C. The next day, the membranes were washed three times with TBST on ice for 10 min before being incubated with HRP-conjugated goat anti-rabbit IgG secondary detection antibodies (Boster Biol Tech) at room temperature for an hour. Subsequently, the membranes underwent three washes in TBST for ten minutes each. Immunostained protein bands were detected using Enhanced Chemiluminescence (ECL) reagent via autoradiography. Band intensities were analyzed using Image-Lab software (Bio-Rad, Hercules, CA, USA). Relative expression was normalized using β-actin as a loading control, and the data are presented as a percentage of reference gene expression.

### ROS detection in DRG sections

Four days after constructing SNI models and administering mitochondrial injection therapy, the DRGs were surgically extracted and sectioned. The levels of reactive oxygen species (ROS) in the DRGs were evaluated using Hydrocyanine-Cy3 staining (ROSstar550, LICOR) and relative fluorescence quantification among the Sham, SNI, SNI with mitochondrial injection therapy (SNI + Mito), and SNI with mitochondrial injection and oxidant scavenger N-acetylcysteine (NAC) treatment (SNI + Mito + NAC) groups (*n* = 3). The isolated DRGs were rinsed in ice-cold PBS-DTPA. Subsequently, they were incubated with 100 µM hydrocyanine-Cy3 in PBS at 37 °C for 30 min, washed once with PBS-DTPA, and then fixed in 4% PFA. The DRGs were then sectioned and scanned using a fluorescence microscope. The ROS levels were assessed by measuring the relative fluorescence intensities.

### Chromatin immunoprecipitation (ChIP) qPCR

The DRG tissue from the Sham, SNI, and SNI with mitochondrial injection therapy (SNI + Mito) groups were subjected to ChIP-PCR. Each group contained 3 samples, with each sample consisting of 3 DRGs (L3/4/5). DRGs were collected and rapidly frozen in liquid nitrogen. The thawed DRGs were dissociated using a Dounce homogenizer with cold TBH buffer (containing protease inhibitors, pH 7.4 Tris, and sucrose). After fixing the cells in TBH containing formaldehyde, glycine was used to quench the fixation reaction before pelleting the cells at 3000 × g for 5 min at 4℃. Nuclei isolation was achieved by incubating the cell pellet in cell lysis buffer, followed by sedimentation of nuclei at low speed. Finally, nuclear lysis buffer was used to lyse nuclei for ChIP analysis after a brief incubation period at room temperature. Then, 850 µL of a dilution buffer containing Tris-Cl (16.7 mM, pH 8), NaCl (167 mM), EDTA (1.2 mM), Triton X-100 (1%), SDS (0.1%), and protease inhibitors was added to the 150 µL nuclear lysis reaction. The chromatin sample (1 mL) was subjected to sonication for 20 min using a Diagenode Bioruptor sonicator with high power and alternating cycles of 30 s on and off. Following sonication, the sheared chromatin was centrifuged at 13,000 × g for 10 min to separate soluble chromatin from insoluble material. The resulting soluble chromatin phase was carefully transferred to a new tube while discarding the insoluble fraction. To remove any nonspecific binding, the soluble chromatin segments were pre-cleared by incubating them with Dynabeads protein A (Invitrogen, catalog number: 10002D) at a temperature of 4℃ for a duration of two hours using a volume of 20 µL. A portion representing one-tenth of the pre-cleared chromatin was reserved as an input control, while the remaining fraction was incubated overnight at 4℃ with antibodies specific for γH2AX (#ab2893, Abcam) and 53BP1 (#ab175933, Abcam). Afterward, an additional volume of Dynabeads protein A, measuring approximately 40 µL was added and allowed to bind for another four hours. The enriched segments were sequentially rinsed with LS buffer (containing 0.1% SDS, 1% Triton X-100, 2 mM EDTA, 150 mM NaCl, 20 mM Tris-Cl at pH 8), HS buffer (containing 0.1% SDS, 1% Triton X-100, 2 mM EDTA, 500 mM NaCl, and a pH of 8), TL buffer (consisting of 1% NP-40, 1% NaDOC, 1 mM EDTA, SDS, LiCl at a concentration of 0.25 M, and Tris-Cl at a concentration of 50 mM with a pH of 8), and TE buffer (composed of 0.1 mM EDTA, 50 mM NaCl, and 10 mM Tris-Cl at pH 8) for five minutes each interval. Chromatin was then released in an elution buffer consisting of 145 µL (containing 1% SDS and 0.1 M NaHCO_3_) for 25 min at 65℃ and 65 × g. Crosslinking was reversed by adding NaCl and EDTA to the solution to achieve final concentrations of 0.2 M NaCl and 0.1 mM EDTA, respectively. The mixture was incubated for 18 h at 65℃ and 65 × g. The decrosslinked samples were purified using QIAGEN MinElute PCR purification columns (QIAGEN, 28,004) following the manufacturer’s instructions. qPCR was performed using Sybr Fast Green Mix (Thermo Fisher, 4,385,610). Occupancy of γH2AX and 53BP1 at each genomic location was calculated using the percent input method.

### RNA-Seq

The DRGs from the Sham, SNI, and SNI with mitochondrial injection therapy (SNI + Mito) groups were subjected to RNA sequencing. Each group contained 2 samples, with each sample consisting of 6 DRGs (L3/4/5 from 2 mice). The Qiagen RNeasy Plus Universal Kit was utilized to extract total RNA from DRGs, following to the manufacturer’s recommended procedure. The Illumina TruSeq Total RNA Sample Prep Kits were utilized to prepare the libraries. The quantification of barcoded libraries was conducted before sequencing on the Illumina Hi-Seq 2000 system. The raw FASTQ data files of paired-end reads were then gathered for further analysis. To align with the UCSC mouse reference genome assembly mm9, TopHat was employed on the generated FASTQ files. The TopHat software utilized the high-throughput short read aligner, Bowtie, for mapping the reads to the reference genome. The aligned reads underwent processing using Cufflinks 2.0.0. Transcript abundance was quantified using fragments per kilobase of exon per million mapped fragments (FPKM). Differential expression analysis comparing different groups was conducted with the Cuffdiff module, and genes exhibiting a p-value < 0.05 were identified as displaying differential expression.

### ChIP-Seq

The L3/4/5 DRGs were isolated from sham and SNI with mitochrondrial injection therapy (SNI + Mito) groups. Each ChIP-seq sample consisted of 18 pooled DRGs from 6 mice per group. The reads from each experiment were combined into single files and utilized for subsequent analyses. For library preparation, 1–5 ng of ChIP (or input) DNA was used. To ensure the retention of library fragments ranging from 300 to 600 bp, gel electrophoresis was employed. Prior to sequencing, libraries were quantified using Qubit (Invitrogen) and assessed for quality using Agilent’s Bioanalyzer. Single-end sequencing with a read length of 36 bp was conducted on the Illumina HiSeq 2000 platform following standard protocols. BWA aligner (samse option) was used to map the sequencing reads to the mouse genome assembly (mm9). Duplicate reads were identified and eliminated using the PICARD tool. The HOMER software was utilized for analyzing γH2AX peaks. To identify differential γH2AX-occupying peaks induced by SNI with mitochondria transfer treatment compared to the sham group, we employed the HOMER software’s findPeaks command with the “region” option specified to manage enriched histone-occupying regions that vary in length. The tag directories for mitochondria transfer treatment and SNI γH2AX ChIP-seq data served as positive and input samples, respectively. Two default parameters, “-size” and “-minDist,” were used for region finding with values of 150 bp and 370 bp, respectively. The peak filtering option was set to 2.5, while the other parameters remained at their default settings. Adjacent peaks within a distance of 700 bp were merged, and regions longer than 1 kb were identified as broad differential γH2AX regions. These broad regions were then extended based on surrounding γH2AX-normalized signals. Bins with normalized signals greater than 1.5 and an edge-to-edge distance less than 1 kb were merged with the broad differential γH2AX regions to generate expanded γH2AX foci. Raw intensities were visualized using the UCSC Genome Browser, with the y-axis standardized for all ChIP-seq runs involving a specific antibody. Aggregation plots of normalized ChIP-seq intensity were generated using the software’s *annotatePeaks.pl* command in the HOMER software and custom R scripts. Publicly available CTCF ChIP-seq datasets provided peak information for aggregate plots, while γH2AX ChIP-seq data determined the binding profile of γH2AX within a ± 2 kb window around these peaks for the aforementioned factors. The y-axis represents the fold-enrichment of DNA bound by γH2AX relative to input DNA in these regions.

### Immunoprecipitation

Immunoprecipitation was conducted according to the instructions provided in a Co-IP Kit (Roche). The L3/4/5 dorsal root ganglions (DRGs) were isolated from sham and SNI with mitochrondrial injection therapy (SNI + Mito) groups. Each Co-IP sample consisted of 18 pooled DRGs from 6 mice per group. The protein extracts from DRGs were prepared following the same procedure as described for Western blot analysis. To eliminate any non-specific binding, the samples were incubated with protein G-agarose on a rotator at 4 °C for 3 h. After centrifugation, the supernatant was transferred to fresh tubes and mixed with 5 µl of anti-γH2AX antibody (#ab81299, Abcam) for 1 h. Subsequently, a homogeneous suspension of protein G-agarose (50 µL) was added and left to incubate overnight at 4 °C on a rotator. Following centrifugation and removal of the supernatant, the beads were washed twice with lysis buffer 1, twice with lysis buffer 2, and once with lysis buffer 3. Protein sample buffer was then added before boiling the samples for 5 min. The presence of CTCF in immunoprecipitated proteins was detected using an anti-CTCF antibody (#ab188408, Abcam) through Western blot analysis.

### Statistical analysis

All data were reported as mean values ± standard deviations (SD). Differences between time points or treatment groups were assessed using two-way analysis of variance (ANOVA) or multivariate analysis of variance (MANOVA). Subsequently, Bonferroni’s post-hoc analysis was conducted to determine the significance between each pair of time points or treatment groups. Statistical analysis was conducted using the Statistical Package for the Social Sciences software (SPSS 15.0 for Windows; SPSS, Chicago, IL). A significance level of *P* < 0.05 was considered statistically significant.

## Results

### MSC-derived mitochondrial injection can effectively promote nerve axon regeneration in mouse sciatic nerve injury (SNI) models

**Fig. 1:**
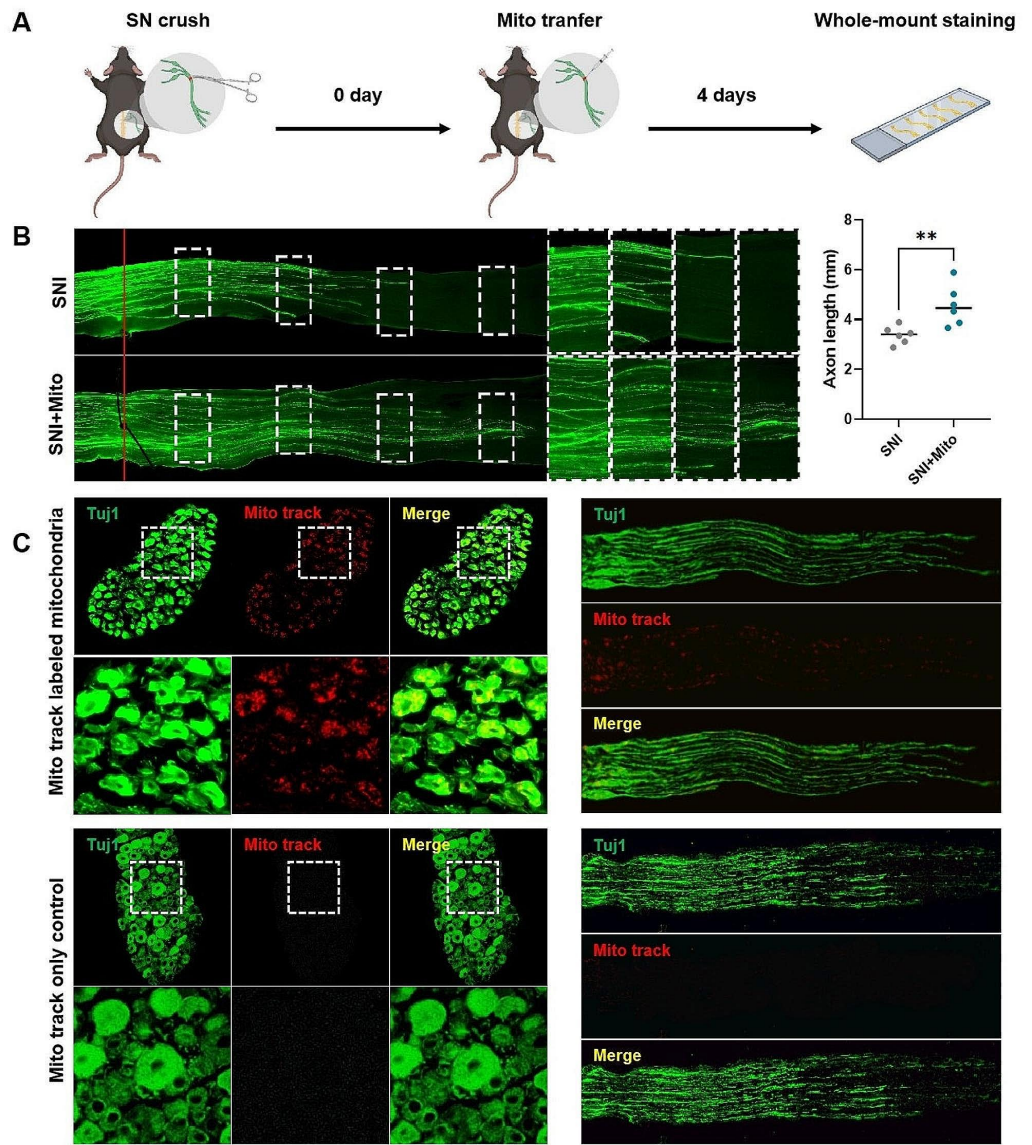
MSC-derived mitochondria injection can effectively promote nerve axon regeneration in a mouse sciatic nerve injury (SNI) model. (**A**) Schematic of the experiment. To construct the SNI model, the right thigh of each mouse was surgically exposed at the mid-thigh level to access the sciatic nerve. The sciatic nerve was then crushed with moderate force using a forceps for a duration of 10 s. For the mitochondrial treatment group, 2 µL of mitochondria derived from 10^6^ MSCs were injected into the crush sites of the injured nerves. The injured sciatic nerves were removed 4 days later. After fixation and clearing, the nerves were whole-mount stained utilizing primary anti-Tuj1 antibodies and fluorescent secondary antibodies, imaging by a fluorescence microscope; (**B**) Representative immunofluorescence images of whole-mount sciatic nerves in the SNI group and SNI with mitochrondria injection therapy group (SNI + Mito) (left) and quantification of the average length of the top 5 axons with the longest regeneration distance in each group (right); (**C**) Fluorescence labeled mitochondrial tracer experiment. In the group injected with Mito-track labeled mitochodria, fluorescence were distributed along the axons (upper right), and were present in the DRGs associated with the injured sciatic nerves (upper left). In the Mito-track only control group, pure Mito-track dye without mitochondria were injected into the injured sciatic nerves, After 4 days, imaging of the sciatic nerve (lower right) and DRG (lower left) revealed minimal fluorescence distribution

Mouse MSCs were used as donor cells to extract mitochondria. The MSC-derived mitochondria were injected into the crush sites of the injured nerves. For each injured nerve in an SNI model, mitochondria extracted from 1 × 10^5^ MSCs were injected. The injured sciatic nerves were harvested 4 days later and were whole-mount stained for Tuj1 (Fig. 1A and Figure S2A). Fluorescence scanning showed that the average length of the top 5 axons with the longest regeneration distance in the mitochondrial treatment group was significantly longer than that in the single SNI control group (Fig. 1B). In the pre-experiment, we also set the time points of 8 and 12 days to assess the length of regenerated axons through whole-mount sciatic nerve staining and to evaluate the functional recovery using Cat Walk test. The whole-mount sciatic nerve staining results showed no significant difference in axon regeneration length on days 8 and 12 (Figure S2B and C). Analysis of the Cat Walk test showed no significant differences between the SNI group and SNI + mito group in terms of printed area (Figure S2D) and swing (Figure S2E) at all three time points. These findings suggest that mitochondrial therapy has a significant promotional effect during early-stage axonal regeneration, although this effect becomes less apparent in later stages, ultimately limiting its translation into improved neural function. Mitochondria extracted from Mito track fluorescently labeled MSCs were injected into the injured nerves. Four days later, staining of the sciatic nerves and dorsal root ganglions (DRGs) staining was performed for observation. The results showed that the fluorescently labeled mitochondria were distributed along the axons and were present in the DRGs associated with the injured sciatic nerves while almost no fluorescent signal was detected in the Mito track only control (Fig. 1C), indicating that injected mitochondria could be retrogradely transferred into DRG neuron somas along nerve axons.

### Mitochondrial injection promotes the expression of regeneration-associated genes (RAGs) in DRGs

**Fig. 2:**
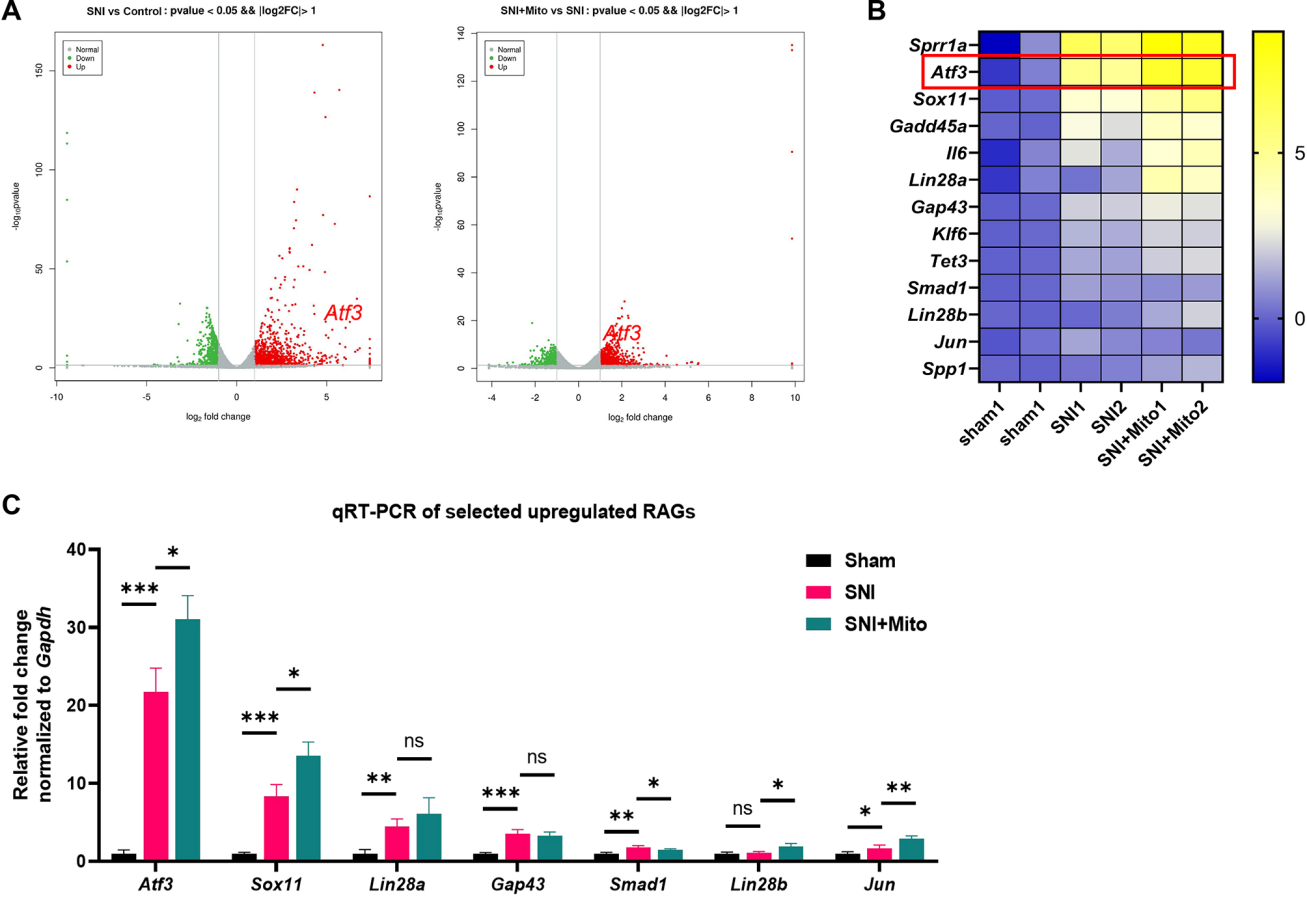
Mitochondrial injection promotes the expression of regeneration associated genes (RAGs) in dorsal root ganglions (DRGs). RNA sequencing and qPCR assays were performed on DRGs among Sham group, sciatic nerve injury (SNI) group and SNI with mitochrondria injection therapy (SNI + Mito) gruop. (**A**) Volcano plot of RNA sequencing. A total of 1221 genes were up-regulated and 630 genes were down-regulated in the SNI group compared with the sham group (left), 777 genes were up-regulated and 440 genes were down-regulated in the SNI + Mito group compared with SNI group (right). (**B**) Heatmap of selected up-regulated RAGs from RNA sequencing; (**C**) qPCR results of the selected RAGs among the sham group, SNI group and SNI + Mito group

Four days after the sciatic nerve crush and mitochondria injection treatment, RNA sequencing and qPCR assays were performed on the DRGs associated with the injured sciatic nerves. The RNA sequencing results showed that 1221 genes were up-regulated and 630 genes were down-regulated in the SNI group compared with the sham group (Fig. 2A, left), among which the expression of several RAGs, such as *Sprr1a, Atf3, and Sox11*, was significantly increased (Fig. 2B). After mitochondria injection therapy, 777 genes were up-regulated and 440 genes were down-regulated in the SNI + Mito group compared with the SNI group (Fig. 2A, right), among which several important RAGs, including *Atf3, sox11* and *Gadd45a*, were further up-regulated (Fig. 2B). While GO and KEGG analysis of these differentially expressed genes did not directly reveal specific connection with axonal regeneration (Figure S3 and S4). qPCR results also showed that the expression of some key RAGs was increased after mitochondrial injection therapy, and *Atf3* was significantly upregulated (Fig. 2C; Figure S5). These results suggest that the mitochondria transferred into injured DRG neurons interact with the nucleus and are involved in the transcriptional regulation of RAGs.

### Atf3 is a crucial upstream target of mitochondrial transfer in regulating the expression of RAGs

**Fig. 3:**
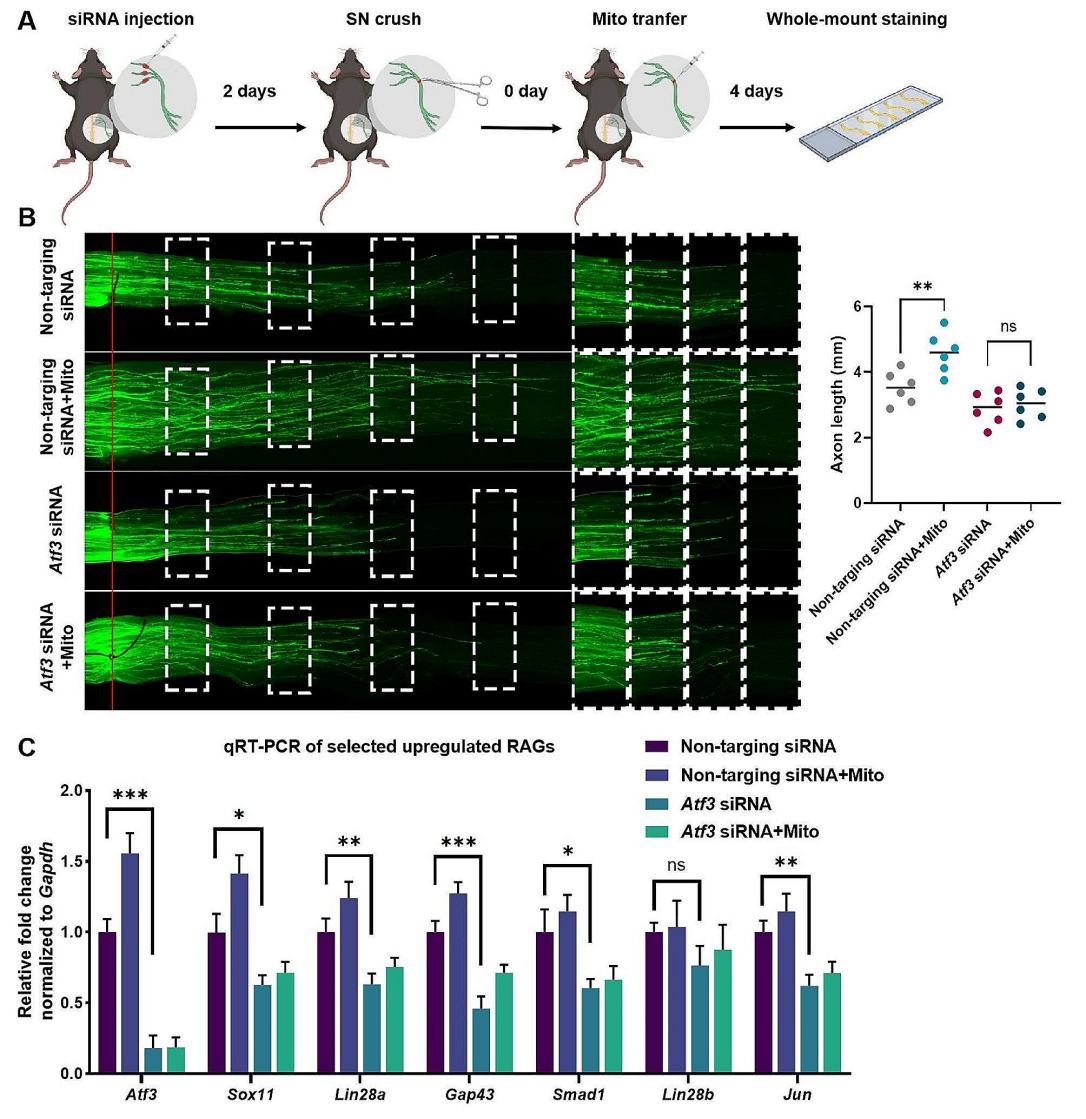
*Atf3* is a key upstream target of mitochondrial transfer in regulating the expression of RAGs. (**A**) Schematic of the experiment. The expression of *Atf3* in DRG cells was knocked down by injection of *Atf3* siRNA in to L3/4/5 DRGs and electrotransfection. The non-targeting siRNA were employed as a control to *Atf3* siRNA. The SNI models were constructed 2 days later. To construct the SNI model, the right thigh of each mouse was surgically exposed at the mid-thigh level to access the sciatic nerve. The sciatic nerve was then crushed with moderate force using a forceps for a duration of 10 s. For the non-targeting siRNA with mitochrondria injection therapy (non-targeting siRNA + Mito) and *Atf3* siRNA with mitochrondria injection therapy (*Atf3* siRNA + Mito) groups, 2 µL of mitochondria derived from 10^6^ MSCs were injected into the crush sites of the injured nerves. The injured sciatic nerves were removed 4 days later. After fixation and clearing, the nerves were whole-mount stained utilizing primary anti-Tuj1 antibodies and fluorescent secondary antibodies, imaging by a fluorescence microscope; (**B**) Representative immunofluorescence images of whole-mount sciatic nerves in non-targeting siRNA, non-targeting siRNA + Mito, *Atf3* siRNA and *Atf3* siRNA + Mito groups (left). Quantification of the average length of the top 5 axons with the longest regeneration distance in each groups (right). (**C**) qPCR of the selected RAGs in DRG tissue from the above 4 groups. The mRNA expression was normalized against GAPDH. Data represent means ± SD. Statistically significant differences are indicated; *n* = 6; *P < 0.05, vs. Control

To investigate the role of mitochondria-promoted *Atf3* in axon regeneration and the expression of RAGs, the expression of *Atf3* in DRG cells was knocked down by siRNA injection and electrotransfection. Two days later, SNI models and MSC-derived mitochondrial injection therapy were constructed. The regeneration length of axons in injured nerves and the gene expression level in DRG cells were measured four days later (Fig. 3A). Tuj1 staining showed that axon regeneration slowed down after *Atf3* knockdown, and the therapeutic effect of mitochondrial injection was reversed (Fig. 3B). The results of qPCR in DRG tissue showed that after *Atf3* knockdown, the expression of *Atf3* in DRG cells decreased significantly, and the expression levels of other important RAGs also decreased (Fig. 3C; Figure S6). This suggests that *Atf3* is a key upstream target for mitochondrial injection therapy to regulate RAG expression.

### Mitochondria promote the expression of ATF3 through ROS-induced DSBs

**Fig. 4:**
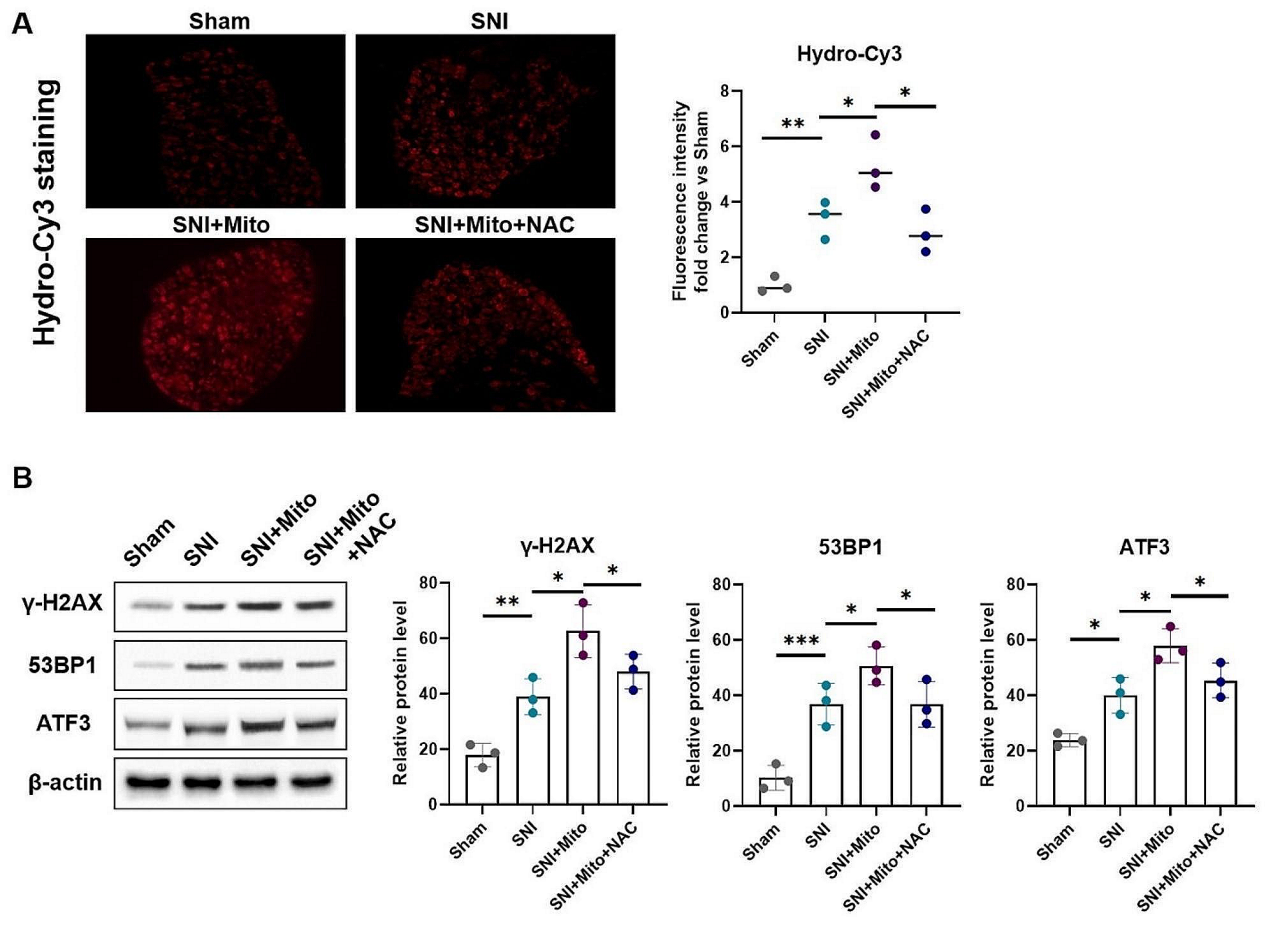
Mitochondria promote the expression of ATF3 through ROS induced DSBs. Four days after constructing SNI models and administering mitochondrial injection therapy, the DRGs were surgically extracted and sectioned. The levels of reactive oxygen species (ROS) in the DRGs were evaluated using Hydrocyanine-Cy3 staining and relative fluorescence quantification among the Sham, SNI, SNI with mitochondrial injection therapy (SNI + Mito), and SNI with mitochondrial injection and oxidant scavenger N-acetylcysteine (NAC) treatment (SNI + Mito + NAC) groups. (**A**) Representative Hydrocyanine-Cy3 staining images of DRG tissue from the Sham, SNI, SNI + Mito and SNI + Mito + NAC groups (left). Relative quantification of Hydrocyanine-Cy3 fluorescence intensities of the above 4 groups. The levels of DNA double strand breaks and ATF3 protein expression in DRGs from the above 4 groups were detect by Western blot. (**B**) Western blot of protein expression levels of DSB markers of γ-H2AX and 53BP1, and ATF3 in DRGs from Sham, SNI, SNI + Mito and SNI + Mito + NAC groups (left). Densitometric analysis of the protein expression of γ-H2AX, 53BP1, and ATF3 among the above 4 groups (right). Protein expression was normalized against β-actin. Data represent means ± SD. Statistically significant differences are indicated; *n* = 3; *P < 0.05, vs. Control

The oxidative respiratory chain at the mitochondrial membrane is an important source of intracellular ROS [40]. To investigate the relationship between ATF3 expression and ROS levels, we employed a Hydrocyanine-Cy3 probe to measure ROS levels in DRG neurons following mitochondrial injection therapy. The results showed a notable increase in ROS content in the SNI + Mito group compared to the SNI group (Fig. 4A). As ROS are a direct factor causing DSBs [41] which play an important role in the expression of early response genes in neuronal injury [42]. We also detected DSB levels in DRG associated with nerve injury following mitochondrial injection therapy. Western blot results revealed a significant increase in the protein expression levels of the DSB markers γ-H2AX and 53BP1 in the SNI + Mito group compared to the SNI group (Fig. 4B). This suggests that the transferred mitochondria exacerbated the extent of DSBs in DRG neurons. After the oxidant scavenger N-acetylcysteine (NAC) was used to antagonize ROS, the effects of mitochondrial injection on promoting both DSB and ATF3 expression were significantly weakened (Fig. 4B). These results suggest that MSC-derived mitochondria increase DSBs through intracellular ROS accumulation, thereby promoting ATF3 expression.

### MSC-derived mitochondria mediate DSBs at the transcription initiation region of the *Atf3* gene

**Fig. 5:**
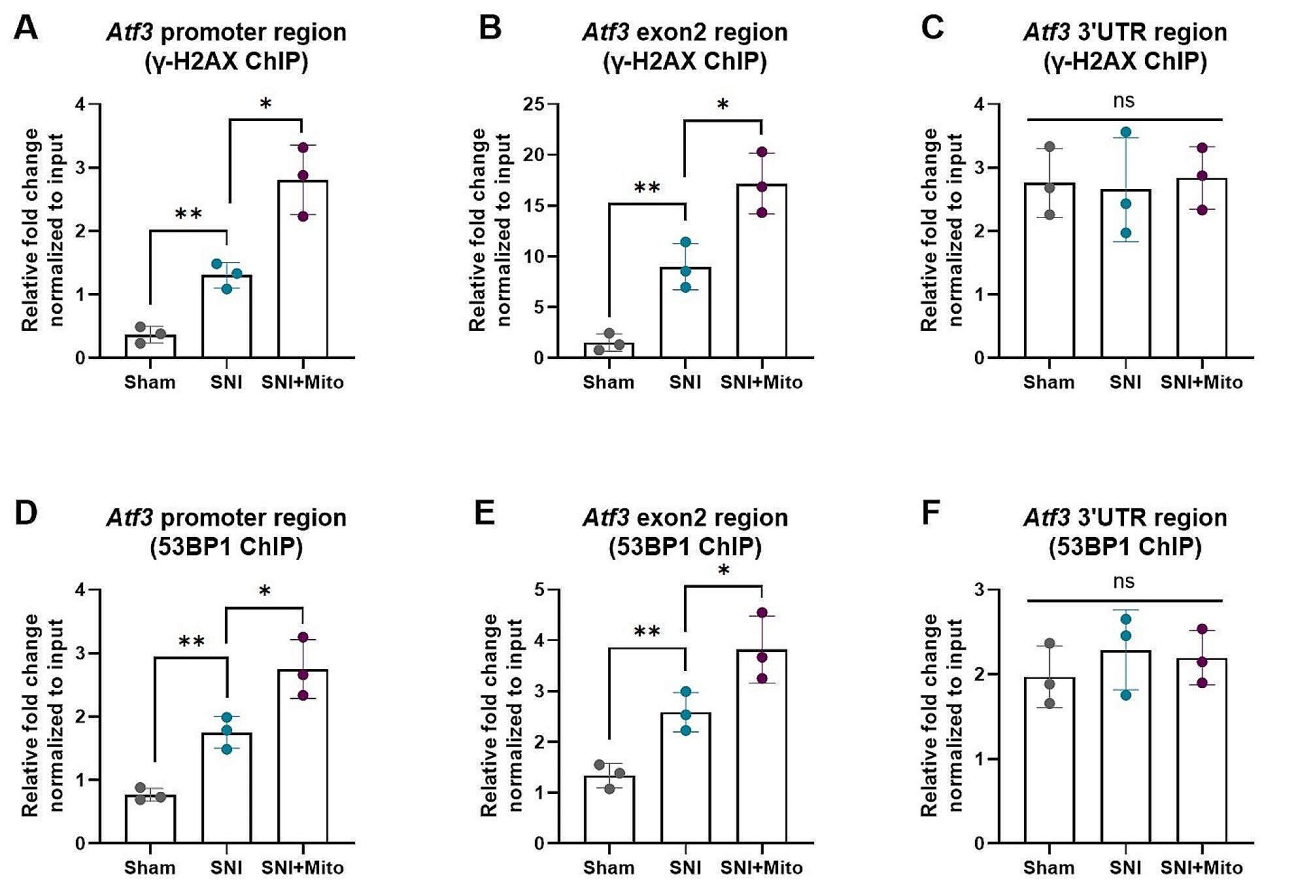
MSC-derived mitochondrial transfer mediates DSBs at the *Atf3* gene transcription initiation region. To detect double-strand breaks (DSBs) in various regions of the *Atf3* gene, the ChIP-PCR assay was employed, utilizing γ-H2AX and 53BP1 antibodies along with primers targeting the *Atf3* promoter, exon 2, and 3’UTR. (**A**-**C**) ChIP-PCR analysis of γ-H2AX binding to the *Atf3* promoter (**A**), exon 2 (**B**) and 3’UTR (**C**) regions in the sham, SNI and SNI with mitochrondrial injection therapy (SNI + Mito) groups. (**D**-**F**) ChIP-PCR analysis of 53BP1 binding to the *Atf3* promoter (**D**), exon 2 (**E**) and 3’UTR (**F**) regions in the sham, SNI and SNI + Mito groups

To determine whether the transcriptional burst of *Atf3* after mitochondrial transfer is related to specific DSB localization, we detected DSBs in different regions of the *Atf3* gene. γ-H2AX and 53BP1 antibodies, as well as primers targeting the *Atf3* promoter, exon 2, and 3’UTR, were utilized for the ChIP-PCR assay. The results showed that DSBs in the *Atf3* promoter and exon 2 regions were detectable in the simple SNI model. Mitochondrial injection therapy further exacerbated the DSBs (Fig. 5A, B, D and E), but there was no significant difference in the 3’UTR (Fig. 5C and F). These results suggest that the up-regulation of *Atf3* expression mediated by mitochondria is related to DSBs in the transcription initiation region of the *Atf3* gene.

### MSC-derived mitochondria-mediated DSBs are close to the CTCF binding site

**Fig. 6:**
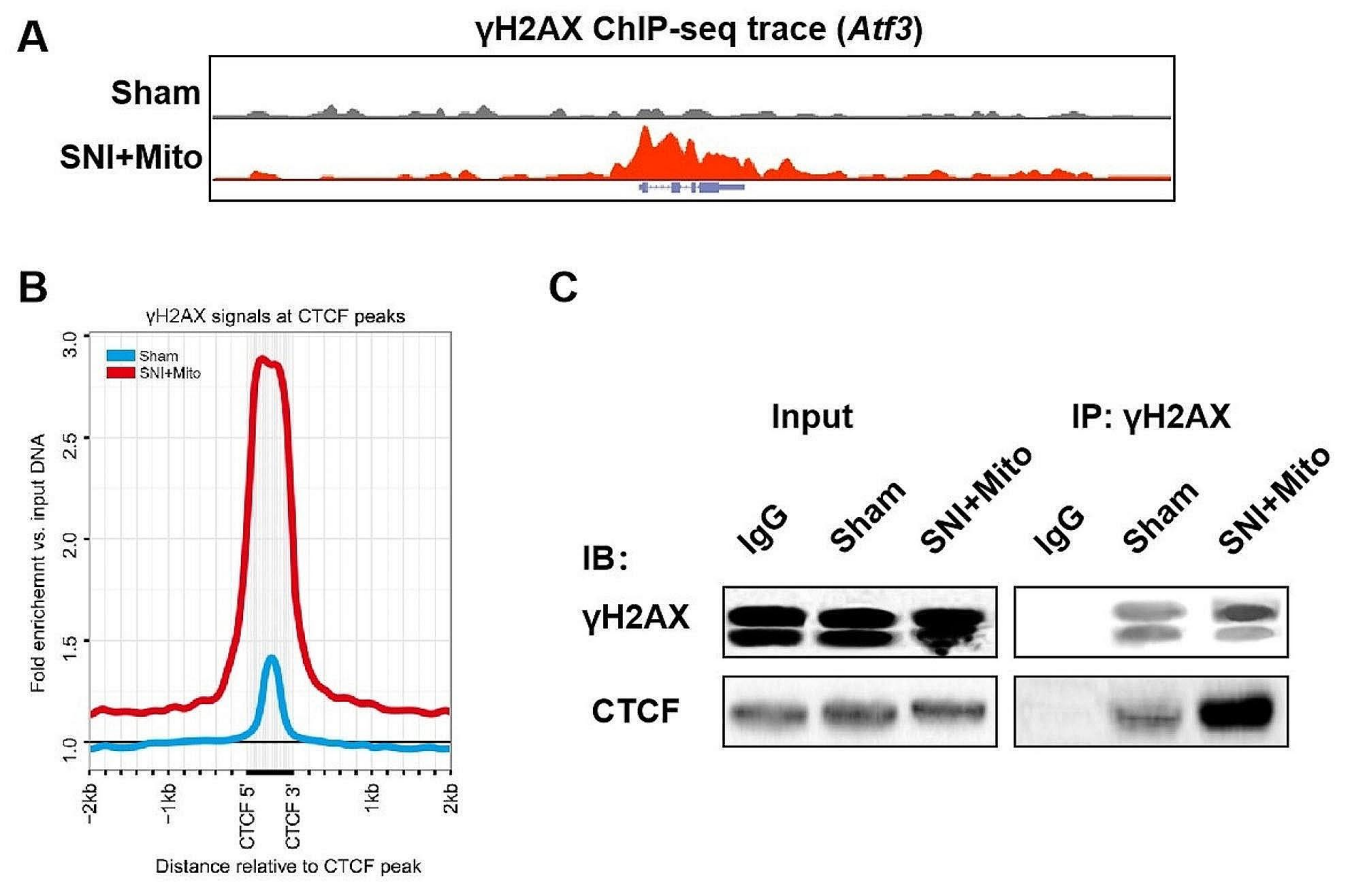
MSC-derived mitochondria-mediated DSBs are close to the CTCF binding site. To investigate the positional correlation between mitochondria-mediated double-strand breaks (DSBs) and CTCF binding sites, dorsal root ganglions (DRGs) were isolated from sham and SNI with mitochrondrial injection therapy (SNI + Mito) groups, and γH2AX ChIP-seq as well as immunoprecipitation were employed. (**A**) UCSC genome browser views denoting the disposition of γH2AX signals at *Atf3* under the sham and SNI with mitochrondrial injection therapy (SNI + Mito). (**B**) The plot denotes the disposition of input-normalized γ-H2AX signals relative to CTCF sites that displayed γ-H2AX peaks in their vicinity. The dashed line denotes the profile of γ-H2AX in the sham group, whereas the solid line indicates γ-H2AX profiles in the SNI with mitochrondrial injection therapy (SNI + Mito) group. (**C**) DRGs from the sham and SNI + Mito groups were lysed and γ-H2AX was immunoprecipitated. The precipitates were analyzed by western blotting with the γ-H2AX and CTCF antibodies

CTCF is an important structural protein involved in the formation of three-dimensional chromatin structures that constrain and control chromatin interactions between topologically associating domains by mediating the topological boundaries through the formation of chromatin rings. To further clarify the reason why the mitochondria-mediated DSBs promote the expression of *Atf3*, we studied the relationship between the location of mitochondria-mediated DSBs and CTCF binding sites. Four days after the sciatic nerve crush and mitochondrial injection, DRG cells were extracted. The γH2AX ChIP-seq results revealed a significant increase in the signal of the *Atf3* gene transcription initiation region (Fig. 6A). Compared with the public CTCF ChIP-seq data (GEO: GSM918727), the γH2AX signal was significantly enriched at the peak of CTCF (Fig. 6B). Co-immunoprecipitation revealed that the interaction between γH2AX and CTCF was significantly enhanced in the mitochondrial therapy group compared to the SNI group (Fig. 6C). These results suggest that the location of MSC-derived mitochondria-mediated DSBs is in the vicinity of CTCF binding sites. Therefore, the topological constraints mediated by CTCF on chromatin interactions may be relieved.

## Discussion

Peripheral nerve injuries commonly occur as a result of car accidents, falls from heights and war injuries, leading to a high disability rate. This significantly increases the social and medical burden [17, 43–45]. Proximal axons have the potential to grow distally after anastomosis of the epineurium or perineurium. However, achieving complete regeneration is challenging, often resulting in residual limb sensor or motor dysfunction [4, 46, 47]. At present, there is a lack of effective methods to promote axonal regeneration in clinical settings.

The phenotypic transformation of Schwann cells (SCs) and the interaction between SCs and injured nerves play pivotal roles in the regeneration of peripheral nerves following injury [48]. Court et al. demonstrated that Schwann cells transfer ribosomes to regenerating axons by supporting local axonal protein synthesis [24, 25]. This finding suggests that the transfer of other organelles, such as mitochondria, from supporting cells to injured nerves may also occur during the process of nerve repair. Additionally, artificial transfer treatments could potentially have therapeutic effects.

Recent studies have found that mitochondrial transfer from bone marrow mesenchymal stromal cells (MSCs) can promote the repair of central nervous system injury [49–51]. In stroke mice, MSC-derived mitochondrial transfer to neurons promotes neuronal survival and enhances cerebral ischemia rehabilitation. In mice with spinal cord injury, injecting purified mitochondria derived from MSCs into the injury site can significantly reduce the apoptosis of motor neurons and promote the repair of spinal cord injury [39]. Kuo et al. revealed that perineurium injection of mitochondria in a sciatic nerve crush injury model can effectively prevent axonal degeneration [52]. However, the therapeutic effect and mechanism of mitochondrial transfer in peripheral nerve injury have not been extensively studied.

In our study, we found that after 4 d of treatment with MSC-derived mitochondrial injections, the length of regenerated axons in the treatment group was significantly longer than that in the control group. Mitochondrial fluorescence was observed in the L4-5 DRGs 4 days after injecting specific fluorescently labeled mitochondria into the SNI model. These results suggest that mitochondria derived from MSCs, when injected into the injured sciatic nerve, can be retrogradely transferred into DRG neuron somas along the nerve axon, promoting the axon regeneration in the injured nerve. Unexpectedly, this therapeutic effect was weakened on days 8 and 12. The analysis of Cat Walk test also revealed negative results, indicating no statistical differences between the SNI group and the SNI + mito group in terms of printed area and swing at all three time points of 4, 8, and 12 days. These findings suggest that mitochondrial therapy has a notable promotional effect during the early stages of axon regeneration, although this effect becomes less pronounced in later stages, ultimately limiting its translation into improved neural function.

Further studies revealed that the expression of various regeneration-associated genes (RAGs) in SNI mouse DRGs was up-regulated compared to the sham group. Additionally, mitochondrial injection treatment further increased the expression of several crucial RAGs, with the *Atf3* gene being notably up-regulated. While GO and KEGG analysis of the differentially expressed genes following the RNA-seq did not drectly reveal specific connection with axon regeneration. This may be attributed to nerve injury and mitochondrial therapy causing extensive and diverse changes in neuronal gene expression rather than simple alterations in processes and pathways related to axonal regeneration. After the knockdown of the *Atf3* gene in the DRG using siRNA, the expression of RAGs was down-regulated and the efficacy of mitochondrial transfer therapy was reversed. Combined with the aforementioned axonal regeneration and functional recovery results, it is postulated that mitochondrial therapy enhances intrinsic motivation for axon regeneration at the transcriptional level. Nevertheless, inhibitory environmental factors may counteract the initial benefits provided by mitochondrial therapy.

The *Atf3* gene is an early response RAG in nerve injury repair [9, 53, 54], and it is also the most strongly expressed transcription factor in the injured and regenerated DRG neurons [9, 55]. ATF3 interacts with many other transcription factors to form a complex that regulates the expression of various downstream genes [56, 57]. *Atf3* is not expressed or is expressed at a very low level in undamaged DRG neurons, but its expression is approximately 30-fold elevated in several hours following axonal injury. This change occurs only in neurons with regenerative capacity [58]. Activation of *Atf3* increases the expression of other RAGs and enhances peripheral nerve regeneration [10]. Conversely, in the *Atf3* KO mouse model of nerve injury, both RAG expression and axonal regeneration decreased [9, 59]. Therefore, *Atf3* acts as a key role in initiating the nerve injury repair program and regulating the expression of other RAGs.

In the nervous system, enhancer-promoter interactions are critical for the expression of early response genes [8, 60, 61]. In the basal state of neurons, enhancers of early response genes are bound by transcription factors such as CREB and are spatially distant from promoters. In the activated state, enhancers are spatially close to promoters and interact with each other to promote gene transcription. At the same time, CREB that binds to enhancers can further recruit transcriptional coactivators, RNA polymerases, etc., to promoters, and further promote transcription [60]. Enhancer-promoter spatial interactions are often regulated by chromatin topology [62–64].

DNA double-strand breaks (DSBs) are crucial biological events that regulate chromatin topology and also play a significant role in the early response stage of neuronal injury. After a cerebral cortex injury, the damaged neurons may trigger the rapid expression of early response genes through the promoter region DSBs, thereby facilitating nerve repair [42]. Reactive oxygen species (ROS) are a direct factor that induces DSBs [41] and are necessary for the regeneration of axons and synapses of damaged sensory neurons [65], while the mitochondrial membrane is an important site for intracellular ROS generation [40].

Our study found that the ROS content and DSB levels in DRG neurons of SNI mice were increased compared to those in the sham group. Additionally mitochondrial transfer therapy further elevated the ROS content and DSB levels. The up-regulatory effect of DSB and *Atf3* mediated by mitochondrial injection therapy was reversed after the addition of ROS antagonists. The presence of DSBs in the promoter and exon 2 of the *Atf3* gene was further confirmed by ChIP-PCR experiments using the antibodies against the DSB markers γH2AX and 53BP1. These results suggest that mitochondrial transfer mediates DSBs in the transcription initiation region of the *Atf3* gene via the ROS accumulation, thereby up-regulating *Atf3* expression. Although the experimental results tend to support ROS accumulation as the primary factor contributing to DSB formation, it is important to note that there are multiple factors that can also cause DSBs, such as activation of topoisomerase III and peroxidation of membrane lipids. Therefore, it should be emphasized that ROS is not necessarily a driver of DSBs.

The potential spatial interaction between the promoter and enhancer is often blocked by the insulating action of the CTCF protein. CTCF is an important structural protein involved in the formation of a three-dimensional chromatin structure that constrains and controls chromatin interactions between topologically associating domains by mediating the topological boundaries through the formation of chromatin rings [66, 67]. In our study, ChIP-seq comparison of γH2AX and CTCF in DRG cells, and the immunoprecipitation of γH2AX and CTCF, showed that the γH2AX signal was more enriched at the peak of CTCF in the mitochondrial transfer treatment group than in the SNI group, and the interaction between γH2AX and CTCF was significantly enhanced. These results suggest that mitochondrial transfer mediated DSBs are localized close to CTCF binding sites. Therefore, the CTCF-mediated topological constraints on chromatin interactions may be relieved, potentially promoting the spatial interaction of the *Atf3* promoter and enhancer.

**Fig. 7:**
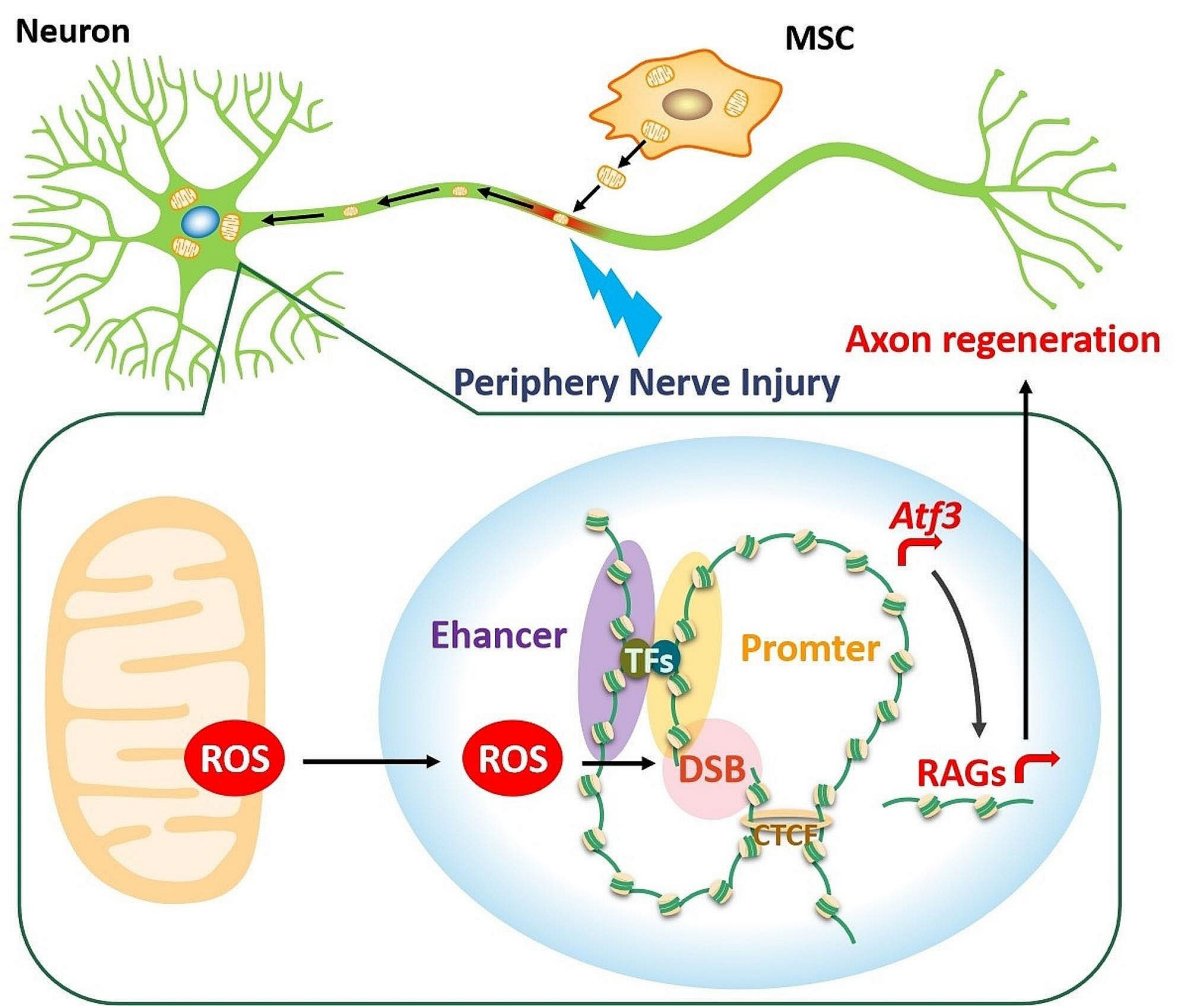
Diagrammatic depiction of the therapeutic effect of MSC-derived mitochondria injection therapy on sciatic nerve injury. MSC-derived mitochondria are retrogradely transferred to DRG neurons via axoplasmic transport, and DSBs at the transcription initiation region of the *Atf3* gene are mediated by ROS accumulation, thereby rapidly releasing topological constraints on chromatin interactions, facilitating spatial interactions between enhancers and *Atf3* promoters, and promoting *Atf3* expression, thus promoting the expression of genes related to regeneration, and finally, promoting axon regeneration

This study also has certain limitations. For the mechanism of mitochondrial treatment, it is possible that mitochondria can be taken up by Schwann cells (SCs) and promote their supportive function in nerve injury repair, indirectly facilitating axonal regeneration. Bai et al. have demonstrated that grafts enriched with MSC-derived mitochondria effectively treated sciatic nerve defects, confirming the uptake of mitochondria by SCs, which promotes proliferation and migration through enhanced energy synthesis, ultimately leading to axonal regeneration [68]. It will be intreseting to investigate the phenomenon and function of mitochondrial uptake in SCs following the mitochodrial injection therapy in SNI models. Additionally, we are currently unable to quantify the proportion of mitochondria that successfully transfer into neurons. It is important to employ more advanced methodologies to assess the mitochondrial transfer efficiency in future investigations.

In conclusion, intraneural injecting MSC-derived mitochondrial is a potential therapy for peripheral nerve injury. The injected MSC-derived mitochondria can be retrogradely transferred to DRG neurons via axoplasmic transport. DSBs at the transcription initiation region of the *Atf3* gene are mediated by ROS accumulation. This process may rapidly release topological constraints on chromatin interactions between the *Atf3* promoter and enhancer, thereby promoting *Atf3* expression and ultimately facilitating axon regeneration (Fig. 7). This study will establish a theoretical foundation for the implementation of mitochondrial transfer therapy in repairing peripheral nerve injuries and identify potential therapeutic targets.

### Electronic supplementary material

Below is the link to the electronic supplementary material.

Supplementary Material 1

Supplementary Material 2

## Data Availability

The datasets used and/or analyzed during the current study are available from the corresponding author on reasonable request.

## Abbreviations

MSC: Bone marrow mesenchymal stromal cell
ATF3: Activating transcription factor 3
DRG: Dorsal root ganglion
ROS: Reactive oxygen species
DSB: DNA double strand break
SNI: Sciatic nerve injury
RAG: Regeneration associated gene
