# Supplementary material for: MSC-derived mitochondria promote axonal regeneration via Atf3 gene up-regulation by ROS induced DNA double strand breaks at transcription initiation region
Source: Cell Commun Signal. 2024 Apr 25;22:240. doi: 10.1186/s12964-024-01617-7 (PMC11046838; doi:10.1186/s12964-024-01617-7)
Supplement: Supplementary file 1 — Supplementary Material 1 [file 12964_2024_1617_MOESM1_ESM.docx]

**1. Identification of MSCs, extraction of mitochondria and construction of SNI models.**

The mouse MSCs were procured from Cyagen Biosciences (Guangzhou, China). The purchased MSCs were the 6^th^ passage, and were cultured to the 10^th^ passage to detect the surface markers and differentiation potential. Flow cytometry showed that CD105, CD73, and CD90 were positive, and CD45, CD34, and HLA-DR were negative (A). Differentiation experiments showed that the MSCs could successfully differentiate into adipoblast, chondroblast and osteoblast (B). The results indicated that the obtained cells were consistent with the expression of MSC surface markers and had the potential of multidirectional differentiation, which confirmed the authenticity and reliability of the cells used.

Mitochondria were isolated from cultured MSCs by mitochondria Isolation Kit (#89874, Mitochondria Isolation Kit for mammalian cells, Thermo Fisher, USA). The structure and morphology of mitochondria were observed by transmission electron microscopy. The results showed that most of the mitochondrial membranes were complete, clear and full in shape (C).

After anesthetizing the mice with isoflurane, the skin of the right lower limb and fascia lata were incised to expose the sciatic nerve, and the middle part of the sciatic nerve was crushed for 10 s with ophthalmic forceps, and the crush site was knoted with nylon thread to mark the injured site (D). 2 μL of the mitochondia (isolated from 10^6^ MSCs) were injected into the sciatic nerve at the crush site using a microsyringe (E)


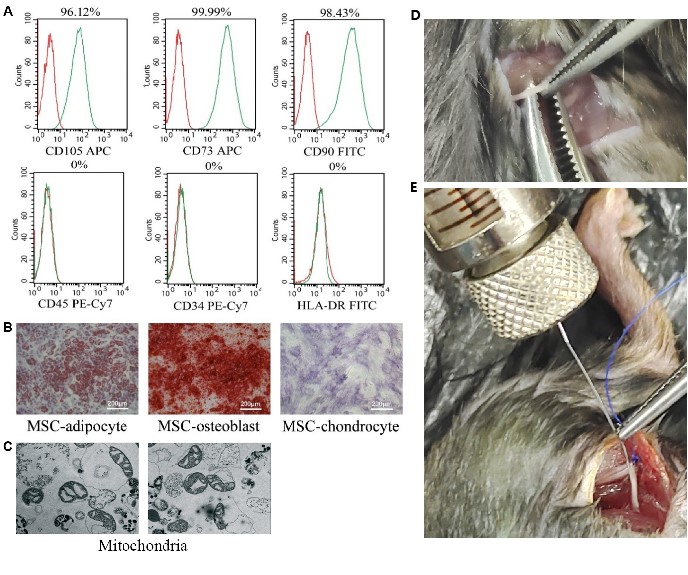


Figure S1. Identification of MSCs, extraction of mitochondria and construction of SNI models

**2. Negative results in the pre-experiment: anxon regeneration on 8 and 12 days; and functional recovery on 4, 8 and 12 days**

In the pre-experiment, we established three observation time points of 4, 8, and 12 days to assess the length of regenerated axons through whole-mount staining. Additionally, we evaluated the functional recovery of the sciatic nerve with or without mitochondrial treatment using the Cat Walk test. The results from whole-mount sciatic nerve staining indicated a significant difference in axon regeneration length on day 4 (A) but no significant difference on day 8 (B) and 12 (C). Furthermore, analysis of Cat Walk test revealed no statistical differences between the SNI group and SNI+mito group in terms of printed area (D) and swing (E) at all three time points. These findings suggest that mitochondrial therapy has a notable promotion effect during early-stage axon regeneration, although this effect becomes less apparent in later stages, ultimately limiting its translation into improved neural function.


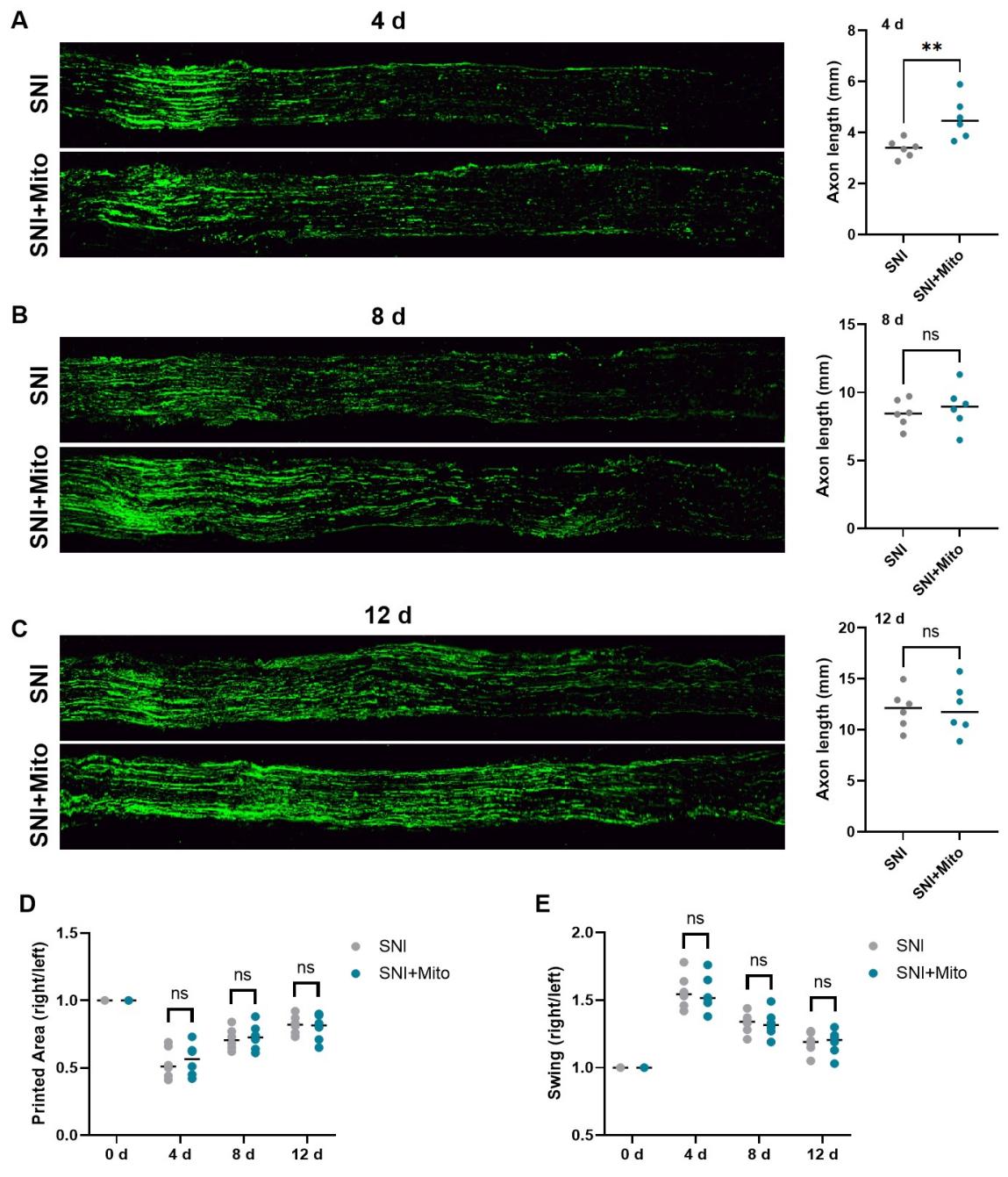


Figure S2. Whole-mount sciatic nerve staining and Cat Walk test in SNI and SNI+mito group on 4, 8 and 12 days.

**3.** **GO enrichment of different expressed genes between SNI and control groups and between SNI+Mito and SNI groups**

Following the completion of RNA-seq, we conducted routine GO and KEGG enrichment analysis on differentially expressed genes. The results of the GO enrichment analysis revealed the most significantly up-regulated (panel A) and down-regulated (panel A) biological processes, cellular components, and molecular functions in the SNI group compared to the Control group. Similarly, panels C and D displayed the most significantly up-regulated and down-regulated biological processes, cellular components, and molecular functions in the SNI+Mito group compared to the SNI group.

**
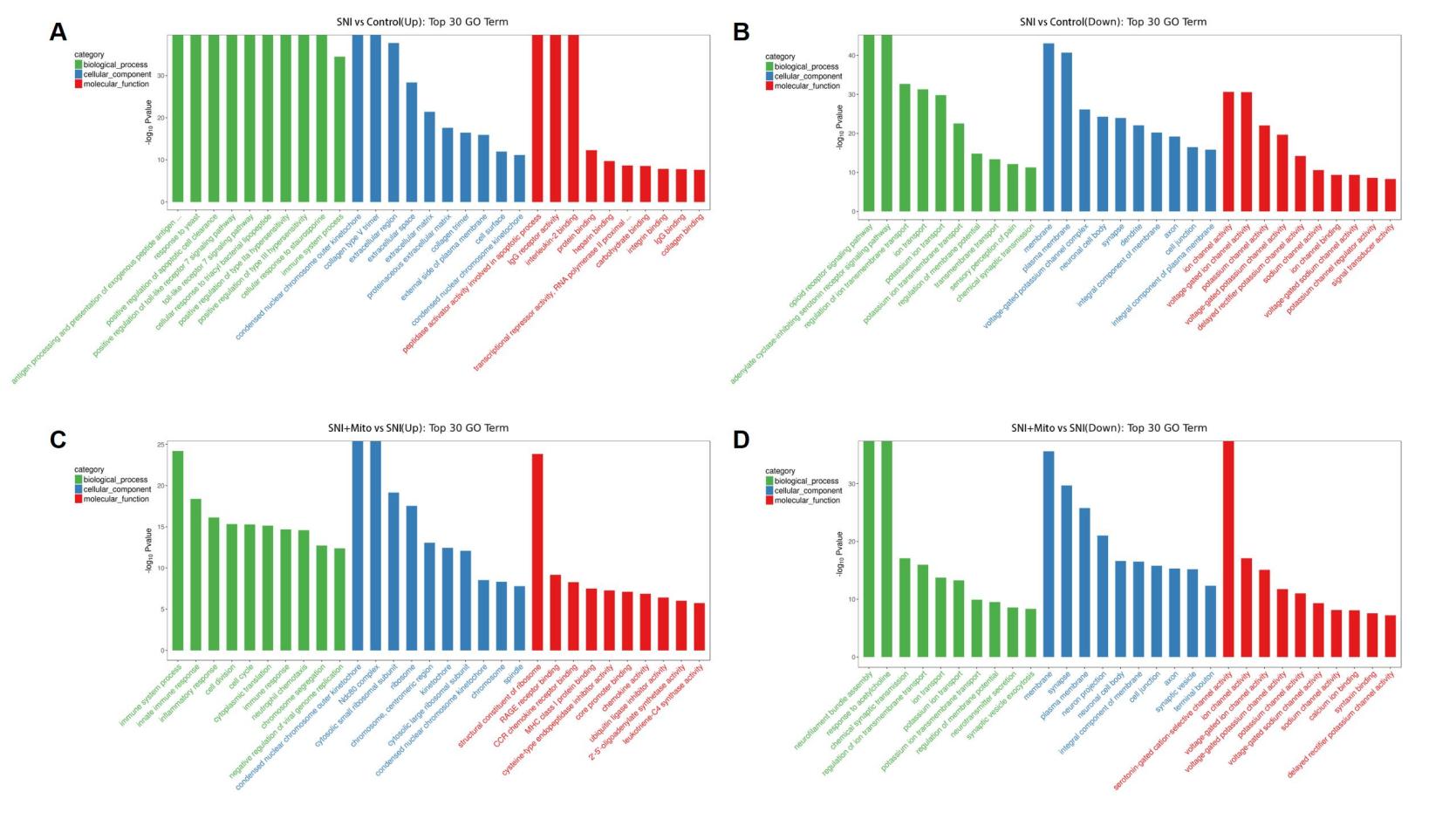
**

Figure S3. GO enrichment of different expressed genes between SNI and control groups and between SNI+Mito and SNI groups

**4.** **KEGG enrichment of different expressed genes between SNI and control groups and between SNI+Mito and SNI groups**

In the results of KEGG enrichment analysis, panel A and B display the most significantly up-regulated and down-regulated signaling pathways in the SNI group compared to the Control group, respectively. Panels C and D exhibit the most significant up-regulated and down-regulated signaling pathways in the SNI+Mito group compared to the SNI group, respectively. However, both GO and KEGG enrichment analyses of differentially expressed genes among these three groups did not directly reveal any specific connections with axonal regeneration. This may be attributed to nerve injury and mitochondrial therapy causing extensive and diverse changes in neuronal gene expression rather than simple alterations in processes and pathways related to axonal regeneration.

**
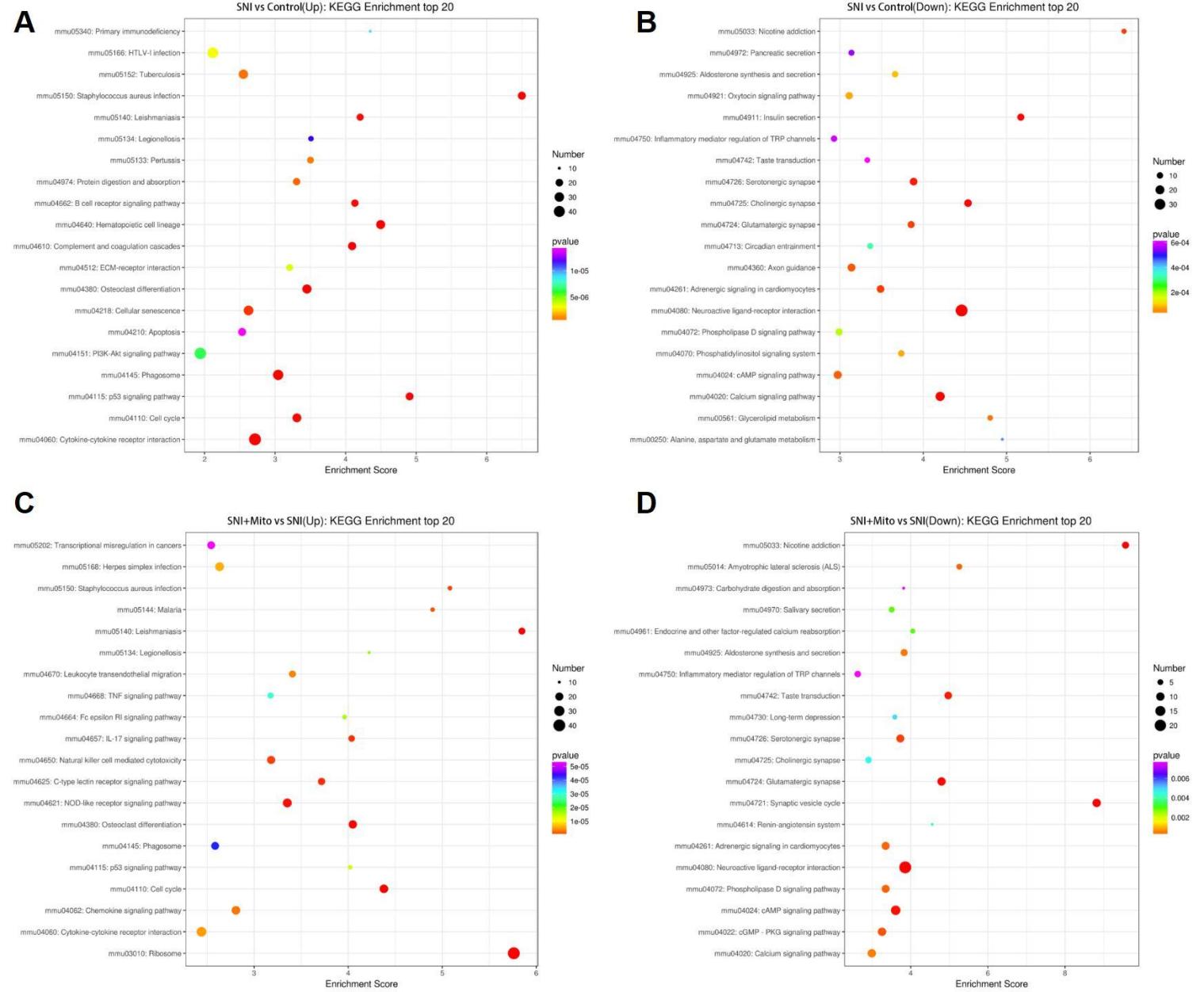
**

Figure S4. KEGG enrichment of different expressed genes between SNI and control groups and between SNI+Mito and SNI groups

**5. Reproductive qPCR result of Figure 2C utilizing β-actin as a control gene.**

To verify the impact of mitochondrial therapy on RAG expression in DRG cells, RNA was extracted from DRGs 4 days after inducing SNI modeling in mice, with or without mitochondrial therapy. The qPCR was employed to assess the levels of selected up-regulated RAGs from RNA-seq data. The qPCR results depicted in Figure 2C demonstrate an increase in the expression of selected key RAGs subsequent to mitochondrial injection therapy, with a significant up-regulation observed for *Atf3*. The control gene used in Figure 2C was *Gapdh*. The subsequent findings yielded consistent results when employing *β-actin* as the control gene, thereby validating the regulatory effect of mitochondrial therapy at RAG expression.


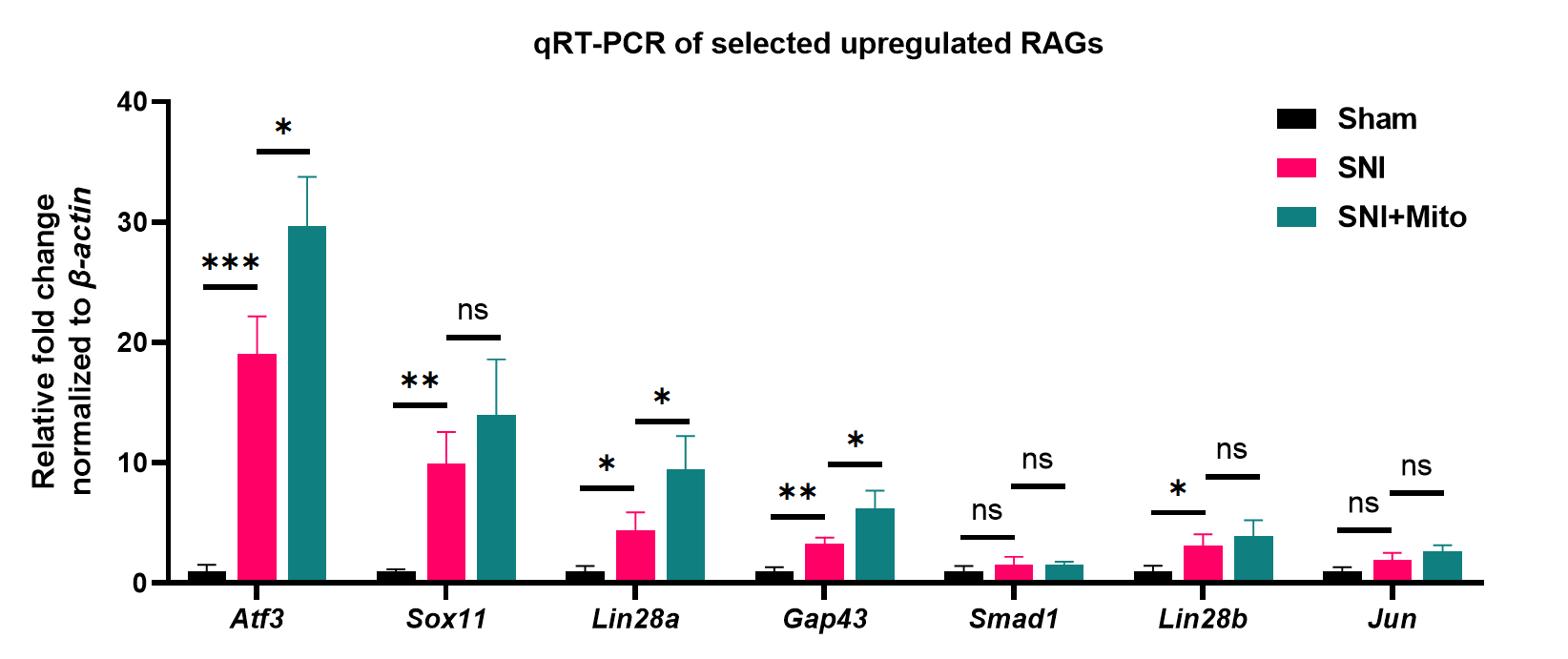


Figure S5. Reproductive qPCR result of Figure 2C utilizing *β-actin* as a control gene

**6. Reproductive qPCR result of Figure 3C utilizing β-actin as a control gene.**

To examine the role of mitochondria-promoted *Atf3* in axon regeneration and the expression of RAGs in SNI models, the expression of *Atf3* in DRG cells was knocked down by siRNA injection and electrotransfection. Then the qPCR was employed to assess the levels of *Atf3* and other selected RAGs. The qPCR results depicted in Figure 3C demonstrate that after *Atf3* knockdown, the expression of *Atf3* in DRG cells decreased significantly, and the expression of other selected RAGs also decreased. The control gene used in Figure 3C was *Gapdh*. The subsequent findings yielded consistent results when employing *β-actin* as the control gene, thereby validating the regulatory effect of *Atf3* on RAGs.


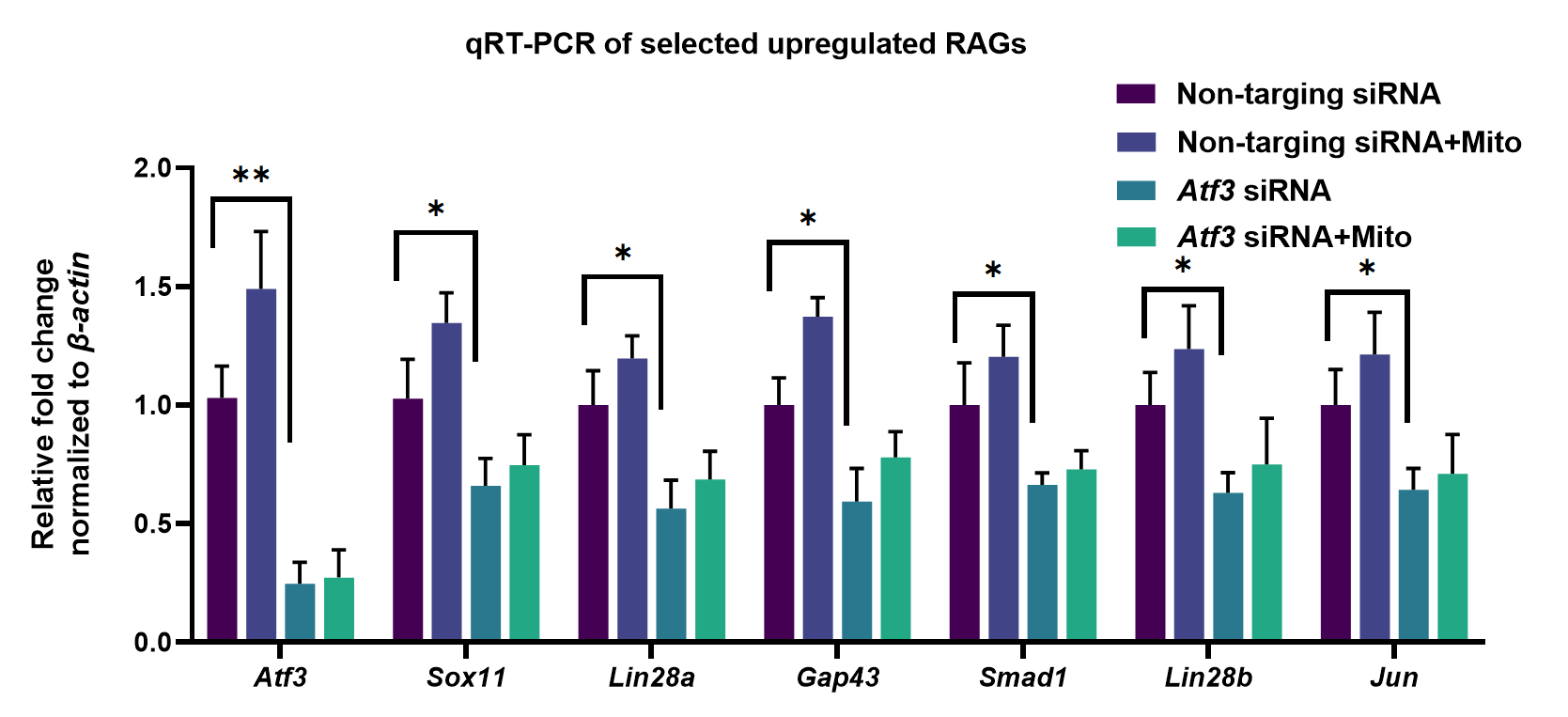


Figure S6. Reproductive qPCR result of Figure 2C utilizing *β-actin* as a control gene
