# Supplementary material for: MSC-derived mitochondria promote axonal regeneration via Atf3 gene up-regulation by ROS induced DNA double strand breaks at transcription initiation region
Source: Cell Commun Signal. 2024 Apr 25;22:240. doi: 10.1186/s12964-024-01617-7 (PMC11046838; doi:10.1186/s12964-024-01617-7)
Supplement: Supplementary file 2 — Supplementary Material 2 [file 12964_2024_1617_MOESM2_ESM.docx]

**Table S1.** The sequences of primers used in this study.

| ***Gene*** | **Forward (5’-3’)** | **Reverse (5’-3’)** |
| --- | --- | --- |
| *Atf3* | CCTCGGAAGTGAGTGCTTCTG | GCAAAATCCTCAAACACCAGTGA |
| *Atf3 promoter* | CAATCCCAGGCTGACGTAATG | GTCTCCACCCACCTTTTGC |
| *Atf3 exon2* | TGGCGAATCTCAGCTCTTCC | ATTGTCCCCTGCCTCTCAC |
| *Atf3 3’UTR* | TATCATTCCCAGCCCTCCAC | AGCCCTTTCCCTCACTTTGT |
| *Sox11* | CAACGCATTGAGTGTGAGCATC | CAGCACCACCTCCTTGTCC |
| *Lin28a* | AATCCATCCGTGTCACTGGC | CTAGCCCACCGCAGTTGTAG |
| *Gap43* | TGGTGTCAAGCCGGAAGATAA | GCTGGTGCATCACCCTTCT |
| *Smad1* | CTCATGTCATTTATTGCCGTGTG | CGCTTATAGTGGTAGGGGTTGA |
| *Lin28b* | GCCTTGAGTCAATACGGGTAAC | AGGGTCTTCCCTTTAGGTCTTC |
| *Jun* | TGTGCCCCAAGAACGTGAC | CCGGGTTGAAGTTGCTGAG |
| *Gapdh* | CCACTTTGTGAAGCTCATTTCCT | TCGTCCTCCTCTGGTGCTCT |
| *β-actin* | GGCTGTATTCCCCTCCATCG | CCAGTTGGTAACAATGCCATGT |
